# The impact of implementing a person-centred pain management intervention on resistance to change and organizational culture

**DOI:** 10.1186/s12913-021-06819-0

**Published:** 2021-12-11

**Authors:** Eva Angelini, Axel Wolf, Helle Wijk, Helena Brisby, Adad Baranto

**Affiliations:** 1 Department of Orthopaedics, Institute of Clinical Sciences, at Sahlgrenska Academy, University of Gothenburg, Gothenburg, Sweden; 2 Institute of Health and Care Sciences at Sahlgrenska Academy, University of Gothenburg, Gothenburg, Sweden; 3 Sahlgrenska Universitetssjukhuset Forskningsenhet ortopedi, Länsmansgatan 28, 431 80 Mölndal, Sweden; 4 Department of Quality Improvement, Sahlgrenska University Hospital, Gothenburg, Sweden; 5 Architecture, Chalmers University of Technology, Gothenburg, Sweden; 6 Department of Orthopaedics, Sahlgrenska University Hospital, Gothenburg, Sweden

**Keywords:** Resistance to change, Organizational culture, Organization, Person-centred care, Implementation, Spine surgery

## Abstract

**Background:**

Resistance to change and organizational culture are essential factors to consider in change management in health care settings. Implementation of structural change remains a challenge. There is a lack of studies providing information on the impact of implementation processes on the organization. The aim of this study was to describe the impact of implementing a systematic change process concerning postoperative person-centred pain management on resistance to change and organizational culture in an orthopaedic spine surgery unit.

**Methods:**

The study was set in an orthopaedic spine surgery unit at a university hospital. Person-centred bundles of care for postoperative pain management of spine surgery patients were developed in co-creation by a multi-professional expert group and implemented throughout the care pathway. The intervention was underpinned by theories on organizational culture and inspired by principles of person-centred care. Quantitative data were collected using the *Resistance to Change Scale* and the *Organizational Culture Assessment Instrument* and analysed using descriptive statistics.

**Results:**

The findings showed a low resistance to change decreasing during the study. The organizational culture shifted from a result-oriented to a formalized and structured culture after the implementation. The culture preferred by the staff was team-oriented and participation-focused throughout the study. The discrepancy between the *current* and *preferred* cultures remained extensive over time.

**Conclusion:**

It is challenging to describe the influence of the development and implementation of a postoperative pain management program on organizational culture as well as in terms of resistance to change, in a complex health care setting. In the current study the unit was under organizational strain during the implementation. Albeit, the important discrepancy between the *current* and *preferred* organizational culture could imply that structural changes aren’t enough when implementing person-centred pain management structures and needs to be combined with relational aspects of change.

**Supplementary Information:**

The online version contains supplementary material available at 10.1186/s12913-021-06819-0.

## Background

In this paper, our focus is on understanding implementation processes by describing the effect of a change process on resistance to change (RTC) and organizational culture (OC) in an orthopaedic surgery setting in a large university hospital. Health care organizations (HCOs) have an obligation to maintain and safeguard high quality through continuous improvement, and so are submitted to constant change to meet demands for improved quality, safety and efficiency. The task of introducing change into overburdened and complex organizations such as in health care is challenging as these settings are large in scale and divided into specialized fields. Moreover, HCOs can develop fatigue with constant changes, leading to resistance to change, and in fact change in health care is often met by resistance [1]. According to Peiperl (2005, p. 348) [2], RTC is “*active or passive responses on the part of a person or group that militate against a particular change, a program of changes, or change in general*”. RTC is mostly described as resistance on an individual basis, but Curt Lewin who introduced the concept in 1930, followed by Coch and French, considered that RTC does not arise from the unique individual but from the context in which the change takes place [3]. According to Lewin, the individual’s behaviour is a product of a complex system, an organization, forming a force field around the individual [4]. This is of relevance in a study like the present where the survey is completed by individuals in a HCO and then aggregated and analysed as one.

An organizational culture can be described as fluid and hard to grasp as it lies under the surface of what is seen and tangible, reflecting the underlying values and shared assumptions within an organization [5]. In health care, the concept has been used for decades [5, 6]. OCs may be contributing factors in poor change implementation. Every organization has its unique culture specific to each lone workplace. The concept of organizational culture originates in the social sciences where the underlying assumption that an organization can be seen as a miniature society and justifies a cultural approach. According to Michie and Williams [7], hospital wards develop their own local OCs. In this study, we were interested in observing a specific ward’s underlying OC during structural change, as Resistance to change and organizational culture may impede organizational development and change in health care [8].

Person-centred care (PCC) is an approach including the patient as an equal partner and stakeholder in health care [9]. It has gained increasing interest as a way to empower patients and improve quality. When operationalizing PCC, deliberate (i.e. planned) strategies are important to consider, as are emergent strategies (i.e. in response to the specific change process) [10]. PCC goes beyond the relationship between patient/person and health care staff to affect intra-professional relations. Moore et al. identify barriers and facilitators when implementing PCC. Barriers may include professionals’ attitudes when implementing PCC as it is easier to employ habitual care routines; facilitators include strong leadership which is important when converting to PCC [11].

Resistance to change, organizational culture and person-centred care are constructs of importance in quality improvement. PCC has shown to have organizational impact; Alharbi et al. [12] identified factors facilitating or obstructing the implementation process including organizational culture, resistance to change and time and rapidity of implementation. Further, Wolf et al. [13] found that health care units reached a higher cultural uniformity after implementing person-centred care. Yet the impact of change in health care regarding person-centred care are not fully explored. Considering the paucity of research on the impact of change processes on RTC and OC. This study sought to understand if and how change processes affect RTC and OC over time in an orthopaedic health care setting.

### Aim

The aim of this study was to describe the impact of implementing a systematic change process concerning postoperative person-centred pain management on resistance to change and organizational culture in an orthopaedic spine surgery unit.

## Methods

### Design

This study has a descriptive, cross-sectional survey design, and is part of a larger research project [14, 15]. The present study consists of a pain management intervention and its’ implementation. The overall change programme design was guided by the integrated Promoting Action on Research Implementation in Health Services (i-PARIHS) framework [16] to explore the impact of the change process on resistance to change and organizational culture. The core constructs in the i-PARIHS framework are innovation, recipient, context and facilitation [16]. The method section will hereby follow the iPARIHS structure as follows.

### Innovation

The overall focus in this study was to give greater attention to patients’ postoperative pain management after elective lumbar spine surgery by including the patients’ narratives and documenting these in medical records to give more coherent care adjusted to each patient. The rationale for the intervention was the lack of a defined structure for dealing with patients’ pain and pain management after planned lumbar spine surgery. This leads to incoherent pain treatment, hence the aim to build structures supporting PCC. An expert group was formed with the purpose of improving postoperative pain management in the unit by implementing a PCC approach. The group members represented health professions involved in postoperative pain management. They had knowledge of PCC but no previous training in PCC. The group comprised representation from: first-line management, orthopaedic surgeons, physicians in training, registered nurses (RN), physiotherapists (PT), and assistant nurses (AN). A total of nine experienced professionals were active in the group simultaneously, representing approximately 15% of the total workforce. The assistant doctors changed over time as they worked in the service for about 6 months; RNs changed likewise due to nurse turnover.

**Fig. 1:**
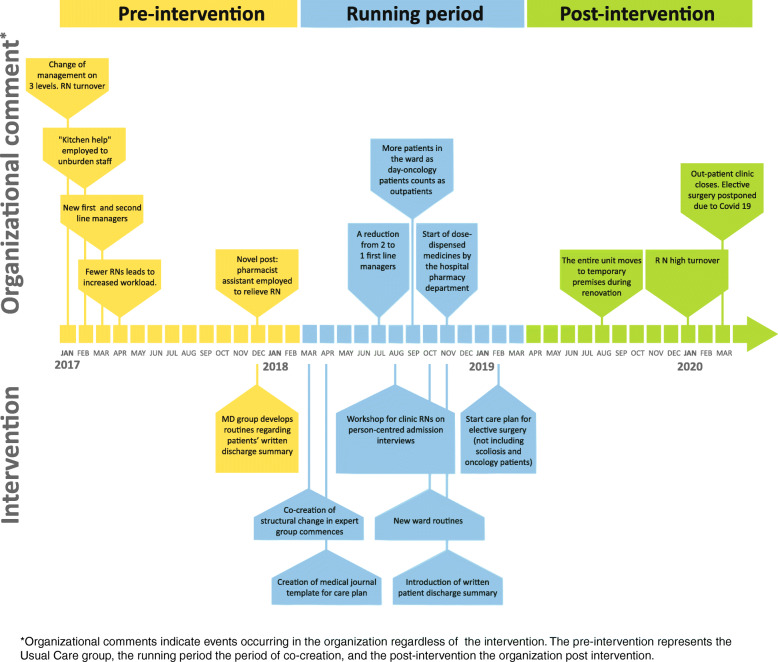
Timeline

The expert group commenced by mapping usual care and the researchers then subsequently developed the intervention together with the expert group. This study extends over a substantial period, March 2017 to March 2020: major organizational events was registered through observation by group members and are shown on a timeline (see Fig. 1). The group met intensively during the co-creation, 10 meetings being held between April and November 2018. In 2019, the group continued to meet regularly to maintain and evaluate the change programme. In addition to the expert group, a group of physicians of diverse seniority and experience met in three sessions in late 2017 to develop routines for patients’ written discharge summaries (required by Swedish legislation since 2012) [17], which had at the time of initiation of the study not been employed in the unit. The group of physicians also established templates specific to the diverse surgeries performed within the unit, to serve as starting points and to be personalized at discharge.

The mapping of the unit revealed the following:

- The documentation in the medical record was fragmented and incoherent, leading to difficulty in following the patient’s reported pain and pain management.
- The daily round was experienced as stressful and seen as inadequate and inefficient by the care staff. The round hampered care as its timing was unpredictable, while decision-making was protracted since assistant doctors were alone while the orthopaedic surgeons were in the operating theatre or in the out-patient clinic. This led to patients not receiving optimal care [14].
- At discharge, no routine existed for patients to receive written information about care given during the hospital stay nor about care after discharge.

The tailoring of the structural change commenced after the mapping, and after multiple sessions in the expert group, a bundle of PCC activities was established, together with implementation strategies for each (Table 1). It presented a care pathway allowing a more articulate focus on patients’ pain management. The timings for implementing each part of the intervention are found in the timeline (Fig. 1). In addition to the expert group, other expertise was employed to develop a documentation template for a care plan with focus on postoperative pain management. The hospital IT and medical records departments were engaged to do this.

**Table 1:** An overview of the intervention and implementation

The pain management intervention	The implementation
Admission interview with the patient/RN regarding pain. The novel routine: The RN obtained the patient’s narrative at the pre-admission visit, including information regarding everyday life and the impact of pain prior to the planned surgery. The narrative was summarized in a care plan, with other clinical information. A tentative PCC plan was written by the RN, including the patient’s recovery goal and expected length of stay. The PCC plan was finalized and updated the evening before planned surgery when the patient was admitted.	Two workshops in autumn 2018 and one in February 2019 were held with RNs in the outpatient clinic, hosted by RN expert in PCC. As new RNs started in the clinic, they received information and training in PCC and documenting patients’ narratives.
Care plan with focus on pain and pain management. The novel routine: Continuous documentation of pain and pain management in the care plan following the guideline. All staff were able to use the plan.	A flowchart to use as a guideline was developed by the RNs in the expert group. RNs in the outpatient clinic and the ward were informed of the use of the care plan, starting in February 2019.
Round routine with explicit roles. The novel routine: Checklist and precise timings for the round. All professions to be present at the round. MD to lead the round according to checklist; RN to document a summary in the care plan.	As all professions were represented in the expert group, relevant professional issues regarding the round emerged in discussions. Professional differences of opinion mostly concerned the timing and the importance of the round. A routine was established by consensus after multiple sessions, and then agreed with relevant first line managers before starting in October 2018.
Written patient discharge summaries. The novel routine: Ward secretaries were assigned to add the template to patients’ journals, and the physician at discharge was responsible for adjusting it to the patient.	Routines for templates were established. Started in November 2018.

### Recipients

The staff comprise physicians, care staff and administrative personnel. All staff working in patient care were asked to participate.

### Context

This study was conducted in a ward specializing in spine surgery at a university hospital in Sweden. In the unit the patients are cared for consists of electively spine surgery patients, spine trauma patients, and orthopaedic oncology patients, including children and adults.

### Facilitation

Each member of the expert group acted as a change agent and facilitator for each phase of the construction and implementation of the change programme, mainly in his or her professional group, thus having the ability to sustain colleagues’ efforts. Facilitators also observed the implementation process, reporting back to the expert group in order to adapt it to the current situation in the unit.

### Theoretical framework

Initially, the Competing Values Framework (CVF) was developed in research studying key factors in effective organizations. This was followed by Quinn and Rohrbaugh further analysing and identifying key factors of effectiveness. The framework consists of two major dimensions of organizational approach: internal focus and integration versus external focus and differentiation: and flexibility and discretion versus stability and control. These form a square divided into four quadrants where each quadrant represents a prominent archetypical organizational characteristic, i.e. culture type. Each quadrant represents basic assumptions, orientations and values characterizing an organizational culture. As the name of the framework indicates, the quadrants compete with each other. The competition is diagonal: thus, the upper left quadrant, *clan*, is in competition with the lower right quadrant, *market*, while the upper right, *adhocracy*, competes with the lower left, *hierarchy*. Below follows a brief explanation of the four culture types:

#### The clan culture (CC)

In this organization, people have a lot in common. Friendships are strong and the unit feels like a big family. The organization promotes teamwork, participation, and consensus.

#### The adhocracy culture (AC)

The organization is dynamic, entrepreneurial, and creative. The organization values individual initiative and freedom.

#### The market culture (MC)

The organization is result-oriented. Reaching goals and gaining a reputation of success are important.

#### The hierarchy culture (HC)

The organization is a formalized and structured workplace. Procedures direct what people do. Work should be efficient and smooth. Stability and results are key.

**Fig. 2:**
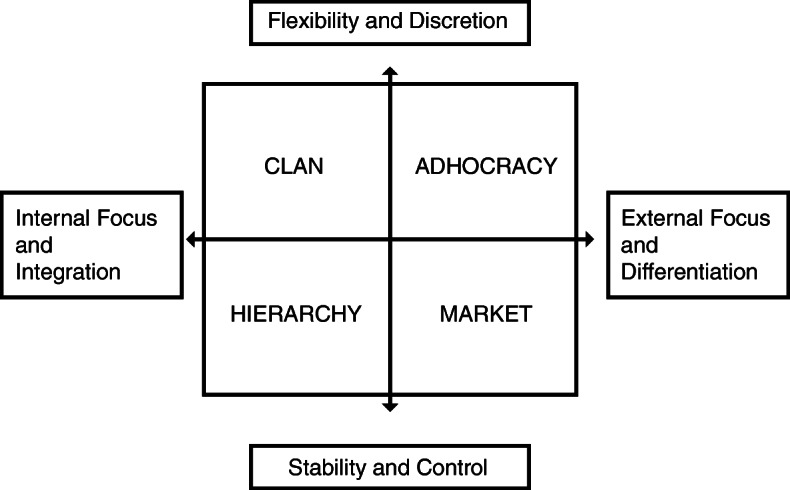
The Competing Values Framework [18]

No cultural type is valued as superior to another; nevertheless, it has been seen that a balanced mixture of OC types is favourable in change processes in HCOs and drives sustainability i.e. the organization’s capacity to sustain change over time [18] (Fig. 2).

### The survey

The questionnaire consists of two instruments: *The Resistance to Change Scale* and *The Organizational Culture Assessment Instrument*.

#### The resistance to change scale

The dispositional Resistance to Change Scale (RTCS) was used to assess the staff reactions to imposed change. In 2003, Oreg [19] developed and validated the scale to establish the existence of disposition to resist change to predict reactions to specific change. The scale was available in a Swedish version. The scale covers the following four factors of an individual’s inclination to resist change:

- Routine seeking (RS)
- Emotional reaction to imposed change (ER)
- Short-term focus (STF)
- Cognitive rigidity (CR)

These factors reflect behavioural, affective, and cognitive aspects of resistance to change.

The behavioural dimension RS reveals people’s inclination to adopt routines. The affective dimension contains ER and STF: ER mirrors the extent of perceived stress and uneasiness the individual experiences when faced with change; STF reflects an individual’s disposition to accept the immediate inconvenience of change in order to obtain a long-term benefit. The cognitive dimension CR relates to dogmatism: change is resisted due to rigidity and a closed mind-set. RTCS scores range from 0 (no resistance) to 6 (maximum resistance) [19].

#### The organizational culture assessment instrument

The Organizational Culture Assessment Instrument (OCAI), based on the CVF developed by Cameron and Quinn, was used to assess OC [18]. The instrument is validated and has been shown to have psychometric validity, albeit weaker with regard to preferred culture [20].

The instrument is widely used, including in health care [21]. The instrument measures the current culture, as well as the preferred culture (i.e. how the respondent would like the unit to be in 5 years in order to be successful) and displays the differences between the current and the preferred cultures. According to Cameron and Quinn, differences larger than 10 points require rapid action [18]. The instrument has an ipsative (forced choice) scale: the respondent divides 100 points between four alternatives in each domain, giving the highest points to the alternative most similar to their organization and decreasing the points as the alternatives differ more and more.

Since there was no Swedish version of the OCAI, a translation was made using the COSMIN checklist for cross-cultural validity [22] (permission granted by the developers of the instrument). The OCAI was translated from English into Swedish by two independent translators, one with and one without expertise in this topic. The Swedish translation was then backtranslated by two independent professional language editors blinded to the original version. Differences were solved by consensus between the translators. Based on this translation, an interdisciplinary expert group was established consisting of an expert in implementation in health care, an orthopaedic surgeon, a specialist nurse, an associate professor, and the authors of the instrument, Kim Cameron and Robert Quinn. Furthermore, one male and one female registered nurse (RN), representing the target population, checked the translation’s coherence in health care environments. There were minor differences between the original and back-translated versions comments which were resolved by the expert group and synthesized into a final Swedish version, where after a pilot study was conducted in an adjacent ward. The pilot was not included in the current study. No major changes were necessary after the pilot study.

### Procedure

Participants received verbal and written information explaining the aim and procedure of the study, together with informed consent forms and self-report questionnaires from the first author (EA). Participation was voluntary. Age and profession were retrieved from the consent forms. Tenure and number of years in profession were collected by the first author (EA). Two reminders were sent by mail 2 and 4 weeks after the due date). Completed questionnaires were returned in allocated binders, with respondents’ anonymity being maintained.

The survey included several measures, with six time points stretching over 2 years and 9 months. The findings were to be presented with baseline, the pre-intervention, (based on four time points exploring the organizational baseline over time: March, June, September 2017, and March 2018), covering the year preceding the start of the change programme. This was to be followed by the period when the change programme was developed, the running period, (September 2018 - one time point), and post-implementation (October to November 2019 - one time point).

Staff working in the ward could respond 1–6 times to the survey. The first time a participant responded, consent and demographic data were collected. At each time point, paper surveys in pre-labelled envelopes were distributed by contact persons, i.e. the ward-coordinator and the secretary in the doctors’ office. The survey went to RNs, ANs, and physicians working 50% or more of a full time at the unit. Managers were not included in the survey.

### Ethics

The Regional Ethical Review Board approved the study (ID number 124–16), which conforms to the principles of the Declaration of Helsinki [23].

### Statistical analysis

Descriptive statistics were presented as mean and standard deviation (SD), or median and interquartile range (IQR) as appropriate. Normality of data was inspected visually by histograms and by the Shapiro-Wilk test.

## Results

In the present study, resistance to change and organizational culture were measured over time parallel to the implementation of structural change. Our findings are presented with regard to the organization as a whole, the staff’s individual scores being aggregated into one overall score.

In total, 353 questionnaires were distributed, of which 198 were returned (56%). The RTCS had 198 correct and valid questionnaires. For the OCAI, questionnaires with miscalculations were excluded, the final number being 143. Of the 198 surveys were all used for the RTCS but only 143 were valid for the OCAI.

### Study population

In total, 119 staff were asked to participate and 81 (68%) did so: demographic data are presented in Table 2.

**Table 2:** Participant demographics

	Frequency	Percent
**Sex**		
Male	36/81	44
Female	45/81	56
Age, mean (SD)	40 (12.7)	
**Age groups**		
20–29	19/81	23
30–39	24/81	30
40–49	16/81	20
50–59	16/81	20
60–69	6/81	7
**Professionals**		
Assistant RN	25/81	31
RN	23/81	28
Assistant PT	1/81	1
PT	3/81	4
Assistant doctor	6/81	7
Resident doctor	9/81	11
Orthopaedic surgeon	14/81	17
**Experience**		
Professional experience, median (IQR) year	9 (4–22)	
Tenure, median (IQR) year	1.2 (0.2–8.5)	

Percentages may not sum to 100 due to rounding

### Timeline

HCOs are complex so we have assembled a timeline to indicate important parameters or events occurring in the unit which imply organizational strain. Our findings are presented in relation to events occurring in the unit (see Fig. 1).

### Resistance to change

In total, 198/353 (56%) complete RTCS questionnaires were returned: the lowest response rate for the different stages was 52% (see Table 3).

**Table 3:** Response rates

	Baseline	Running period	Post-intervention
**Frequency**	132/239	34/52	32/62
**Percent**	55	65	52

The findings show a stable, rather low, resistance to change, slightly declining over time. The mean RTCS scores, as well as all of four of its factors, reduced over time. CR gave the highest scores, revealing some degree of dogmatism where the organization resists change due to rigidity and closed mind-sets. On the other hand, STF had the lowest scores, reflecting a disposition to accept an immediate inconvenience of change in order to obtain a long-term benefit. Further, ER and RS clustered close to the mean RTCS scores (see Table 4).

**Table 4:** RTCS scores

	Group					
	Baseline		Running period		Post-intervention	
	Mean	SD	Mean	SD	Mean	SD
**RTCS**	2.62	0.46	2.68	0.56	2.46	0.56
**Routine seeking**	2.49	0.57	2.56	0.50	2.40	0.61
**Emotional reaction**	2.59	0.79	2.80	0.91	2.40	1.16
**Short-term focus**	2.11	0.78	2.14	0.88	1.89	0.70
**Cognitive rigidity**	3.32	0.68	3.24	0.80	3.18	0.70

### Organizational culture

In total, 143 (40.5%) complete OCAI questionnaires were returned: 96 from the baseline group, 24 from the running period, and 23 post-intervention. The OCAI measures three facets of OC: the current or “the now”, the preferred, and the discrepancy between the current and the preferred.

### The current culture

At baseline, the *market* culture is narrowly the dominant one, representing goal achievement and success, though the *clan* and *hierarchy* cultures score almost as highly. *Adhocracy* received the lowest score. Post-intervention, *hierarchy* is the more dominant culture, representing a dominance of structure, procedures, efficiency and predictability. *Hierarchy* is closely followed by *market*, then *clan* and lastly *adhocracy*. Thus, the current culture displays a balanced mixture of cultures over time with a slight emphasis placed on results and profitability.

### The preferred culture

At baseline, *clan* culture is the dominant preferred culture, representing friendship, teamwork and participation. *Clan* was followed by the *adhocracy* and *hierarchy* cultures. The *market* culture received the lowest score. Post-intervention, *clan* remains dominant, followed by *hierarchy* and *adhocracy*. The *market* culture remains the lowest scoring.

### The discrepancy between the current and preferred cultures

At baseline, there is a discrepancy between the dominant current culture, *market* (27.61, Table 5), and the dominant preferred culture, *clan* (34.94, Table 6). In the post-intervention group, the discrepancy is now between the dominant current culture, *hierarchy* (30.06, Table 5), and the dominant preferred culture, *clan* (33.58, Tables 5 and 6). Further, the *market* culture displays the largest discrepancy between the current and preferred measures (Tables 5 and 6). Thus, there is a persistent and obvious gap between current and preferred cultures, with the discrepancy between *market* and *clan* persisting over time.

**Table 5:** OCAI The current measure

	Group					
	Baseline		Running period		Post-intervention	
	Mean	SD	Mean	SD	Mean	SD
**Clan now**	26.98	12.29	21.49	12.98	22.36	12.37
**Adhocracy now**	19.10	6.04	16.60	5.85	18.36	7.32
**Market now**	27.61	9.91	28.73	14.10	29.21	13.31
**Hierarchy now**	26.24	8.82	33.40	10.54	30.06	12.65

**Table 6:** OCAI The preferred measure

	Group					
	Baseline		Running period		Post-intervention	
	Mean	SD	Mean	SD	Mean	SD
**Clan Pref**	34.94	9.26	36.56	13.83	33.58	12.09
**Adhocracy pref**	23.08	5.17	22.75	8.45	22.29	5.25
**Market pref**	19.29	6.21	16.90	8.95	19.06	5.97
**Hierarchy pref**	22.68	6.45	23.31	12.96	25.00	8.54

## Discussion

The current study describes the effect of an implementation of a systematic change process concerning postoperative person-centred pain management on resistance to change and organizational culture in an orthopaedic spine surgery setting. These findings show multiple organizational events indicating organizational strain, a rather low but stable and slightly descending RTC, and a stable and balanced mix of OC, with nonetheless a considerable discrepancy between the current and preferred cultures.

Person-centred care has seen a remarkable increase over recent years: for example, in the current orthopaedic ward, Angelini et al. found staff wanting increased opportunities for adapting care to each patient to give a more personalized approach [15]. The amplified demand for PCC entails structural change, but this does not automatically lead to cultural change. OC is frequently talked about as the overarching factor when addressing quality in health care. On the other hand, it is commonly identified as the villain that needs to be tackled in order to solve organizational issues. Mannion and Davies imply that the influence of OC can be overemphasized [24]. In the present study, we looked at OC change over a 2.5- year period, a substantial time in an HCO. The timeline lists multiple organizational events leading to organizational strain. Despite these events (changes in management, staff turnover etc.), RTC and OC basically remained stable (Table 3). The events presented as organizational strain on the timeline are not exceptional for HCOs in general, these being involved in a constant demand for change with a focus on quality. Health care organizations are subject to multifaceted challenges from both internal and external contexts. Nilsen and Bernhardsson highlight the importance of, but difficulty in, addressing the influence of the external context on change [25]. In the current research, it is likewise difficult to distinguish the impact of both internal and external contexts.

Our findings reveal a rather low RTC with an overall reduction over time, indicating an increased readiness for change. The shift to a dominant culture of *hierarchy,* with clearer roles being a prerequisite for higher quality, is consistent with the findings of Wolf et al. who found that a culture of routines and structure increased when implementing structured person-centred care. In contrast to our findings, Jones and Van de Ven found that RTC increased over time leading to a weaker commitment to the organization and perception of reduced organizational effectiveness [26]. Likewise, it has been found that planning, routines and goal setting (market and hierarchy cultures i.e. stability and control) appeared to increase RTC [8]. Further, Naldermirci et al. studied deliberate and emergent strategies when implementing person-centred care [10]. In our study, the expert group developed emergent strategies as clinical practice was continuously adapted in the unit during the co-creation of structural change. Jones and Van de Ven found that supportive leadership could reduce RTC [26]. One known factor in achieving change is engaged and strong leadership [11, 27]. Management was represented in the expert group but communication with and engagement from executive management outside the expert group was feeble.

In the current study, OC was observed in order to detect a possible influence on OC when implementing PCC structures. The discrepancy between the current and preferred OCs was extensive and persistent over time, with the dominant preferred culture being the *clan* culture. The discrepancy implies a strong and persistent desire for culture change, from stability and control to a more person-oriented culture. These results further demonstrate that under organizational strain the preferences for the OC remain stable. This persistent gap might indicate that the implementation itself not systematically applied person-centredness for staff throughout the change process. The importance of a systematical approach has been shown in Ekman et al. [28]. Did the implementation merely attain a structurally based change and not a relational change? A greater focus on relational change might have been achieved with an intensified emphasis on person-centredness within the expert group and increased facilitation within the organization. The i-PARIHS framework has facilitation as a major construct, but the framework lack a person-centred construct. We believe that the framework may benefit from adding person-centredness, between facilitators as well as with the staff. Facilitations needs to impact both structure and relations within the organization by dialogue and partnership building, i.e. person-centered facilitation. Thus, the framework might reach further and attain a more sustainable impact. Facilitating change is complex, as facilitators must balance the groups’ different perspectives and attitudes regarding resistance to change. And while resistance, and consequently RTC, is generally seen as something negative, a growing body of research questions whether this is always the case [29, 30]. A lack of RTC could hinder sustainability in quality as no scrutiny of the change would take place. This could be potentially harmful as, without sustainability, continuity could not be achieved. Oreg sought through three studies to link performance and RTC, his findings revealing that resistant individuals were good at routine and monotonous tasks but not at non-routine tasks. This gave stability to routine work [31] which could be valuable in HCOs, where routine work is a large and fundamental part of their work. Further, Amarantou et al. [32] searched for factors affecting RTC and found that it was influenced by both individual and organizational factors, mediated by employee participation in the decision-making.

### Limitations and strengths

A methodological limitation to the study could be the use only of a quantitative method, as the nature of the organizational culture is complex, this construct might have demanded a mixed methods design: future studies should include qualitative methods in order to get a fuller picture of a specific unit. Nevertheless, patients in the unit were interviewed in focus group interviews prior to the intervention, the result of Angelini et al. [14] was discussed with and guided the expert group in the current study. However, patients were not part of the expert group. On the other hand, a strength in our study is the careful choice of questionnaires, only validated instruments previously used in health care being selected [19, 33].

Our findings emerged from a relatively low participation rate but survey reluctance is an increasing phenomenon [34]. The OCAI was chosen as it was a validated and well-established instrument albeit not translated into Swedish. The translation described in the current study was challenged with the culture aspect of health care in the origin context of the instrument (USA) and Sweden, being two different cultures. Another issue was the generic culture aspect of the instrument not developed for health care in particular. In addition, health care in Sweden is in large non-profit therefore production and market aspects are infrequent concepts. Additionally, some staff found the OCAI time-consuming, adding to existing stress, as staff felt they did not have time to fill out the questionnaire during working hours. Further, the OCAI’s ipsative scale was a problem as we had to exclude > 50 questionnaires due to miscalculations. The difference in the final numbers of RTCS and OCAI is problematic and might have weakened the interpretation of the result as the RTC score is based on a larger base than the OC score. An online version would have prevented this problem, as this would have indicated miscalculations immediately. The design also entailed some respondents’ having answered several times, perhaps limiting the breadth of data. However, our goal was to survey the organization as a whole and not professionals, with their groups and subcultures.

Further, the participants median tenure in the unit were 1.2 years. In general, younger staff and staff in training change units, whereas, senior staff tend to be less mobile, here displayed in the vast IQR (0.2–8.5) years.

## Conclusions

Complexity is inherent in large health care organizations and in the current study multiple events occurred during the study period plausibly affecting the results. Our findings suggest that the process of implementing structural change in an orthopaedic spine surgery care unit slightly reduces resistance to change but maintains the discrepancy between current and preferred organizational cultures. The amplified demand for PCC entails structural change, but as seen in the current study structural change is not enough and does not automatically lead to cultural change. Relational change is likewise required. The clinical relevance and importance of the current study within quality improvement is that implementation of change needs to combine the structural as well as the relational aspect of change.

## Supplementary Information

**Additional file 1.**

**Additional file 2.**

## Data Availability

The datasets generated and/or analysed during the current study are not publicly available due the risk this poses to the confidentiality of participants. However, de-identified data is available from the corresponding author on reasonable request.

## Abbreviations

RTC: Resistance to change
OC: Organizational culture
HCOs: Health care organizations
PCC: Person-centred care
i-PARIHS: The Integrated Promoting Action on Research Implementation in Health Services
RN: Registered nurses
PT: Physiotherapists
AN: Assistant nurses
CVF: Competing Values Framework
CC: The clan culture
AC: The adhocracy culture
MC: The market culture
HC: The hierarchy culture
RTCS: Resistance to Change Scale
RS: Routine seeking
ER: Emotional reaction to imposed change
STF: Short-term focus
CR: Cognitive rigidity
SD: Standard deviation
IQR: Interquartile range
