# Supplementary material for: The impact of implementing a person-centred pain management intervention on resistance to change and organizational culture
Source: BMC Health Serv Res. 2021 Dec 11;21:1323. doi: 10.1186/s12913-021-06819-0 (PMC8665601; doi:10.1186/s12913-021-06819-0)
Supplement: Supplementary file 1 — Additional file 1. [file 12913_2021_6819_MOESM1_ESM.docx]

**Instructions**

The OCAI consists of six items, each with four alternatives. Divide 100 points among these four alternatives, depending on the extent to which each alterative is similar to your own organization. Give a higher number of points to the alternative that is most similar to your organization. For example, on item 1, if you think that alternative A is very similar to your organization, alternatives B and C are somewhat similar, and alternative D is hardly similar at all, you might give 55 points to A, 20 points to B and C, and 5 points to D. Just be sure that your total equals 100 for each item.

Note that the left-hand response column for the instrument is labelled “Now”. These responses mean that you are rating your organization as it is currently, not as you would like it to be or as you hope it would be. Complete that rating first. The right-hand column refers to your organization as you think it should be in five years in order to be spectacularly successful, achieve its highest aspirations, become an outstanding example of high performance, outstrip the currently stated goals, or become the benchmark for your industry.

Rate the organization in its current state using the “Now” column. Then complete the instrument the second time using the “Preferred” column.

| **1.** | **Dominant Characteristics** | **Now** | **Preferred** |
| --- | --- | --- | --- |
| **A** | The organization is a very personal place. It is like an extended family. People seem to share a lot of themselves. |  |  |
| **B** | The organization is a dynamic entrepreneurial place. People are willing to stick their necks out and take risks. |  |  |
| **C** | The organization is very results-oriented. A major concern is with getting the job done. People are very competitive and achievement-oriented. |  |  |
| **D** | The organization is a very controlled and structured place. Formal  procedures generally govern what people do. |  |  |
| **Total:** |  |  |  |

| **2.** | **Organizational Leadership** | **Now** | **Preferred** |
| --- | --- | --- | --- |
| **A** | The leadership in the organization is generally considered to exemplify mentoring, facilitating, or nurturing. |  |  |
| **B** | The leadership in the organization is generally considered to  exemplify entrepreneurship, innovation, or risk taking. |  |  |
| **C** | The leadership in the organization is generally considered to  exemplify a no-nonsense, aggressive, results-oriented focus. |  |  |
| **D** | The leadership in the organization is generally considered to exemplify coordinating, organizing, or smooth-running eﬃciency. |  |  |
| **Total:** |  |  |  |

| **3.** | **Management of Employees** | **Now** | **Preferred** |
| --- | --- | --- | --- |
| **A** | The management style in the organization is characterized by teamwork, consensus, and participation. |  |  |
| **B** | The management style in the organization is characterized by individual risk taking, innovation, freedom, and uniqueness. |  |  |
| **C** | The management style in the organization is characterized by hard-driving competitiveness, high demands, and achievement. |  |  |
| **D** | The management style in the organization is characterized by  security of employment, conformity, predictability, and stability in relationships. |  |  |
| **Total:** |  |  |  |

| **4.** | **Organization Glue** | **Now** | **Preferred** |
| --- | --- | --- | --- |
| **A** | The glue that holds the organization together is loyalty and mutual  trust. Commitment to this organization runs high. |  |  |
| **B** | The glue that holds the organization together is commitment to  innovation and development. There is an emphasis on being on the cutting edge. |  |  |
| **C** | The glue that holds the organization together is an emphasis on achievement and goal accomplishment. |  |  |
| **D** | The glue that holds the organization together is formal rules and  policies. Maintaininga smooth-running organization is important. |  |  |
| **Total:** |  |  |  |

.

| **5.** | **Strategic Emphases** | **Now** | **Preferred** |
| --- | --- | --- | --- |
| **A** | The organization emphasizes human development. High trust, openness, and participation persist. |  |  |
| **B** | The organization emphasizes acquiring new resources and creating new challenges. Trying new things and prospecting for opportunities are valued. |  |  |
| **C** | The organization emphasizes competitive actions and achievement. Hitting stretch targets and winning in the marketplace are dominant. |  |  |
| **D** | The organization emphasizes permanence and stability. Eﬃciency, control and smooth operations are important. |  |  |
| **Total:** |  |  |  |

| **6.** | **Criteria of Success** | **Now** | **Preferred** |
| --- | --- | --- | --- |
| **A** | The organization deﬁnes success on the basis of development of  human resources, teamwork, employee commitment, and concern for people. |  |  |
| **B** | The organization deﬁnes success on the basis of having the most unique or newest products. It is a product leader and innovator. |  |  |
| **C** | The organization deﬁnes success on the basis of winning in the  marketplace and outpacing the competition. Competitive market leadership is key. |  |  |
| **D** | The organization deﬁnes success on the basis of eﬃciency.  Dependable delivery, smooth scheduling and low-cost production are critical |  |  |
| **Total:** |  |  |  |

**Worksheet for scoring the OCAI**

|  | **NOW** | **Preferred** |
| --- | --- | --- |
| **1A** |  |  |
| **2A** |  |  |
| **3A** |  |  |
| **4A** |  |  |
| **5A** |  |  |
| **6A** |  |  |
| **Sum** (of A responses) |  |  |
| **Average**  (sum divided by 6) |  |  |

|  | **Now** | **Preferred** |
| --- | --- | --- |
| **1B** |  |  |
| **2B** |  |  |
| **3B** |  |  |
| **4B** |  |  |
| **5B** |  |  |
| **6B** |  |  |
| **Total** (of B responses) |  |  |
| **Average**  (sum divided by 6) |  |  |

|  | **Now** | **Preferred** |
| --- | --- | --- |
| **1C** |  |  |
| **2C** |  |  |
| **3C** |  |  |
| **4C** |  |  |
| **5C** |  |  |
| **6C** |  |  |
| **Total** (of C responses) |  |  |
| **Average**  (sum divided by 6) |  |  |

|  | **Now** | **Preferred** |
| --- | --- | --- |
| **1D** |  |  |
| **2D** |  |  |
| **3D** |  |  |
| **4D** |  |  |
| **5D** |  |  |
| **6D** |  |  |
| **Total** (of C responses) |  |  |
| **Average**  (sum divided by 6) |  |  |
