# Supplementary material for: The impact of implementing a person-centred pain management intervention on resistance to change and organizational culture
Source: BMC Health Serv Res. 2021 Dec 11;21:1323. doi: 10.1186/s12913-021-06819-0 (PMC8665601; doi:10.1186/s12913-021-06819-0)
Supplement: Supplementary file 2 — Additional file 2. [file 12913_2021_6819_MOESM2_ESM.html]

Resistance to Change Scale - Prof. Shaul Oreg


# Resistance to Change Scale Questionnaire

|  |  |
| --- | --- |
| Age |  |
| Gender | Choose One Male Female |
| Family status | Choose One Single Married Divorced Widowed |
| Religion | Choose One Christian Muslim Jewish Hindu Buddhist Other |
| Country of birth |  |

**Instructions:**  
Listed below are several statements regarding one's general beliefs and attitudes towards change.  
Please indicate the degree to which you agree or disagree with each statement by circling the appropriate number on the scale next to it.

| # | Statement | Strongly disagree | Disagree | Inclined to disagree | Inclined to agree | Agree | Strongly agree |
| --- | --- | --- | --- | --- | --- | --- | --- |
| 1 | I generally consider changes to be a negative thing. | 1 | 2 | 3 | 4 | 5 | 6 |
| 2 | I'll take a routine day over a day full of unexpected events any time. | 1 | 2 | 3 | 4 | 5 | 6 |
| 3 | I like to do the same old things rather than try new and different ones. | 1 | 2 | 3 | 4 | 5 | 6 |
| 4 | Whenever my life forms a stable routine, I look for ways to change it. | 1 | 2 | 3 | 4 | 5 | 6 |
| 5 | I'd rather be bored than surprised. | 1 | 2 | 3 | 4 | 5 | 6 |
| 6 | If I were to be informed that there's going to be a significant change regarding the way things are done at school, I would probably feel stressed. | 1 | 2 | 3 | 4 | 5 | 6 |
| 7 | When I am informed of a change of plans, I tense up a bit. | 1 | 2 | 3 | 4 | 5 | 6 |
| 8 | When things don't go according to plans, it stresses me out. | 1 | 2 | 3 | 4 | 5 | 6 |
| 9 | If one of my professors changed the grading criteria, it would probably make me feel uncomfortable even if I thought I'd do just as well without having to do any extra work. | 1 | 2 | 3 | 4 | 5 | 6 |
| 10 | Changing plans seems like a real hassle to me. | 1 | 2 | 3 | 4 | 5 | 6 |
| 11 | Often, I feel a bit uncomfortable even about changes that may potentially improve my life. | 1 | 2 | 3 | 4 | 5 | 6 |
| 12 | When someone pressures me to change something, I tend to resist it even if I think the change may ultimately benefit me. | 1 | 2 | 3 | 4 | 5 | 6 |
| 13 | I sometimes find myself avoiding changes that I know will be good for me. | 1 | 2 | 3 | 4 | 5 | 6 |
| 14 | I often change my mind. | 1 | 2 | 3 | 4 | 5 | 6 |
| 15 | I don't change my mind easily. | 1 | 2 | 3 | 4 | 5 | 6 |
| 16 | Once I've come to a conclusion, I'm not likely to change my mind. | 1 | 2 | 3 | 4 | 5 | 6 |
| 17 | My views are very consistent over time. | 1 | 2 | 3 | 4 | 5 | 6 |

You did not respond to question number:
